# Role of right ventricular–pulmonary arterial coupling assessed by echocardiography to predict adverse outcomes in patients with acute pulmonary embolism

**DOI:** 10.1186/s43044-024-00554-7

**Published:** 2024-09-09

**Authors:** Amir Mostafa, Mahmoud Medhat, Hossam Alhosary, Wassim Amin

**Affiliations:** 1 Cardiovascular Department, Cairo University, Cairo, Egypt; 2 National Heart Institute, Cairo, Egypt

**Keywords:** Pulmonary embolism, RV–PA coupling, Echocardiography, TAPSE/PASP ratio

## Abstract

**Background:**

Pulmonary embolism (PE) is a lethal type of venous thromboembolic disease. Right ventricular (RV) failure is not an uncommon complication of PE leading to higher adverse outcomes. The tricuspid annular peak systolic excursion/pulmonary artery systolic pressure (TAPSE/PASP) ratio as a surrogate for RV–pulmonary artery coupling has proven to be among the predictor of clinical outcomes in multiple patient groups. We evaluated in this study the role of TAPSE/PASP ratio in predicting adverse clinical outcomes in patients with acute PE.

**Results:**

Among patients with established diagnosis of acute PE admitted to the coronary care unit, echocardiography was done within 12 h of admission and TAPSE/PASP ratio was calculated. The patients were followed during hospitalization and after discharge for 3 months for development of adverse outcomes including rehospitalization due to heart failure, recurrent PE and mortality. A total of fifty-five consecutive patients were recruited with mean age 58.3 ± 6.9 years and nearly equal male-to-female ratio. The mean ratio of TAPSE/PASP was 0.479 ± 0.206. In-hospital and 3-month follow-up showed that 10.9% needed rehospitalization with heart failure, 14.5% developed recurrent pulmonary embolism, and mortality was 9.1%. TAPSE/PASP ratio was significantly lower among the patients who developed adverse outcomes. TAPSE/PASP ratio was among the independent predictors of rehospitalization with heart failure, recurrent pulmonary embolism but not mortality at 3-month follow-up. TAPSE/PASP ratio predicted rehospitalization with heart failure at a cutoff point ≤ 0.325, with 100% sensitivity and 79.6% specificity, and predicted recurrent pulmonary embolism at a cutoff point ≤ 0.325, with 75% sensitivity and 78.7% specificity.

**Conclusion:**

TAPSE/PASP ratio is a noninvasive tool that can predict the development of early adverse outcomes in patients with acute PE including rehospitalization with heart failure and recurrent pulmonary embolism.

## Background

Pulmonary embolism (PE) is a lethal type of venous thromboembolic disease. It is considered the third among the cause of cardiovascular mortality with in-hospital mortality rates between 5 and 10% [1, 2].

Right ventricular (RV)–pulmonary artery (PA) coupling refers to relation between the RV afterload and the RV contractility. Chronic conditions associated with increased pulmonary artery (PA) pressure and RV afterload provide time for the RV contractility to increase to try to maintain the RV–PA coupling. This is not the case for acute conditions as acute PE, so this leads to failure of this balance causing acute RV failure that is associated with increased adverse clinical outcomes mainly rehospitalization and mortality [3].

RV failure complicates 30% of acute PE patients leading to 5% in-hospital mortality. This emphasizes the importance of early detection of RV dysfunction in these patients [4].

The main method for assessing the RV–PA coupling is the right heart catheterization (RHC). Multiple research has been conducted to find noninvasive modalities to evaluate RV–PA coupling including echocardiographic measurement of the ratio between tricuspid annular peak systolic excursion and pulmonary artery systolic pressure (TAPSE/PASP) [3].

The predictive value of TAPSE/PASP ratio has been evaluated in multiple patient groups including patients with pulmonary hypertension, heart failure, valvular heart disease and post-cardiac surgery [5–9].

Few studies addressed the predictive value of TAPSE/PASP ratio in patients with acute PE. We aimed in this study to evaluate the predictive role of TAPSE/PASP to detect adverse clinical outcomes in acute PE patients.

## Methods

The study was cross-sectional analytical prospective including fifty-five patients with confirmed diagnosis of acute PE admitted to the intensive care unit of the cardiovascular department of Kasr Alainy Hospital, Cairo University, in the period between January 2023 and January 2024.

Eligible participants included patients ≥ 18 years admitted with normotensive acute PE according to the latest European Society of Cardiology guidelines [10]. Exclusion criteria included previous PE, history of RV dysfunction, history of tricuspid valve disease or tricuspid valve replacement, PE due to malignancy, PE with hemodynamic instability and technical difficulties in assessing RV–PA coupling by echocardiography.

After obtaining informed consent [11], history and physical examination data were collected. Laboratory workup and 12-lead electrocardiogram were done. Within 12 h of admission, transthoracic echocardiography was done using a vivid machine equipped with a transthoracic 2.5 MHz transducer. Echocardiographic parameters assessed included left ventricular (LV) size, LV contractility, right atrial (RA) size, RV size and PASP. RV systolic function was evaluated by RV S wave velocity, RV fractional area changes (RV-FAC) and TAPSE. TAPSE/PASP ratio was used as a measure for RV–PA coupling.

All patients were followed during their hospital admission for their clinical status and after discharge for 3 months to detect the development of short-term outcomes including rehospitalization with heart failure, recurrent pulmonary embolism and mortality.

Statistical analysis was conducted using Statistical Package for the Social Science (SPSS) 28th edition. Mann–Whitney's and Chi-square tests were used to evaluate nonparametric and parametric variables, respectively. Univariate and multivariate regression analyses were used to detect the predictors of development of adverse outcomes. A receiver operator characterized curve (ROC) was constructed to assess the sensitivity, specificity and area under the curve (AUC) of TAPSE/PASP ratio to predict adverse outcomes and to set optimal cutoff point for development of these adverse outcomes. *P* value < 0.05 was used to prove statistical significance.

## Results

Basic demographic data of patients are illustrated in Table 1; nearly half of patients were males with mean age 58.3 ± 6.9 years. All laboratory workup was within normal except for elevated levels of HbA1C, as illustrated in Table 2.

**Table 1:** Basic demographic and clinical data of the patients enrolled with acute pulmonary embolism

Demographic and clinical parameters	Frequency (%)/Mean ± SD
Age	58.3 ± 6.9
Male gender	28 (50.9)
Body mass index (BMI) (kg/m^2^)	30.06 ± 2.27
Heart rate	75 ± 12.6
Respiratory rate (breath/min)	14.1 ± 1.48
Mean arterial pressure (mmHg)	105.8 ± 16
Smoking	24 (43.6)
Diabetes mellitus	32 (58.2)
Hypertension	38 (69.1)
Family history of coronary artery disease	13 (23.6)

**Table 2:** Laboratory data of the patients enrolled with acute pulmonary embolism

Laboratory parameters	Mean ± SD
Hb (g/dL)	13.67 ± 1.5
Platelets (10^3^/μL)	233.07 ± 51.8
TLC (10^3^/μL)	8.46 ± 2.8
Creatinine (mg/dL)	1.004 ± 0.203
Urea (mg/dL)	36.05 ± 5.9
Na (mEq/L)	138.58 ± 2.86
K (mEq/L)	4.187 ± 0.43
INR	0.995 ± 0.106
HbA1C (mg/dL)	7.19 ± 1.65

*Hb* hemoglobin, *TLC* total leukocytic count, *HbA1C* glycosylated hemoglobin, *INR* International Normalized Ratio

Echocardiographic data revealed normal mean LV dimensions and contractility, normal mean RA dimensions, normal mean RV dimensions and normal RV systolic function. The mean ratio of TAPSE/PASP was 0.479 ± 0.206 (Table 3).

**Table 3:** Echocardiographic profile of the patients enrolled with acute pulmonary embolism

Echocardiographic parameters	Mean ± SD
LVEDD (mm)	4.84 ± 0.6
LVESD (mm)	3.2 ± 0.55
Ejection fraction %	60.44 ± 7.09
Right atrial size (cm)	3.244 ± 0.34
Right ventricle 1 size (cm)	3.99 ± 1.1
Right ventricle 2 size (cm)	3.76 ± 0.84
Right ventricle 3 size (cm)	6.65 ± 0.86
Right ventricle-FAC %	39.66 ± 9
IVC diameter (cm)	1.88 ± 0.26
DTI S’ (mm/s)	9.32 ± 2.5
TAPSE (mm)	18.2 ± 4.6
PASP (mmHg)	41.8 ± 12.4
TAPSE/PASP ratio	0.479 ± 0.206

*DTI* Doppler tissue imaging, *FAC* fractional area change, *LVESD* left ventricular end systolic volume, *LVESD* left ventricular end diastolic volume, *IVC* inferior vena cava, *PASP* pulmonary artery systolic pressure, *TAPSE* tricuspid annular plane systolic excursion

At 3-month follow-up, mortality accrued in 9% of the patients (Table 4).

**Table 4:** Adverse outcomes of the patients enrolled with acute pulmonary embolism

Adverse outcomes	Frequency (%)
Rehospitalization with heart failure	6 (10.9)
Recurrent pulmonary embolism	8 (14.5)
Mortality	5 (9.1)

Binary logistic regression was executed for factors predicting occurrence of adverse events (Tables 5, 6, 7), where TAPSE/PASP ratio predicted rehospitalization with heart failure, with an odds ratio of 0.964. Also, TAPSE/PASP ratio predicted occurrence of recurrent pulmonary embolism, with an odds ratio of 0.99, while it couldn’t predict mortality.

**Table 5:** Binary logistic regression for factors predicting rehospitalization with heart failure in the patients enrolled with acute pulmonary embolism

Parameters	Sig.	Odds Ratio	95% CI	
			Lower	Upper
Age	0.229	0.854	0.661	1.1
Sex	0.231	7.78	0.270	22.39
Diabetes mellitus	0.130	23.3	0.397	136.55
Mean arterial pressure	0.433	1.05	0.926	1.19
TAPSE/PASP	0.046	0.964	0.931	0.99

*PASP* pulmonary artery systolic pressure, *TAPSE* tricuspid annular plane systolic excursion

**Table 6:** Binary logistic regression for factors predicting recurrent pulmonary embolism in the patients enrolled with acute pulmonary embolism

Parameters	Sig.	Odds ratio	95% CI	
			Lower	Upper
Age	0.772	0.975	0.822	1.16
Sex	0.234	0.247	0.025	2.46
Diabetes mellitus	0.065	0.087	0.006	1.16
Mean arterial pressure	0.857	1.006	0.942	1.07
TAPSE/PASP	0.02	0.99	0.981	0.998
INR	0.545	0.043	0.0	114.78

*PASP* pulmonary artery systolic pressure, *TAPSE* tricuspid annular plane systolic excursion

**Table 7:** Binary logistic regression for factors predicting mortality in the patients enrolled with acute pulmonary embolism

Variable	Sig.	Odds ratio	95% CI	
			Lower	Upper
Age	0.996	0.001	0.00	–
Sex	0.995	1.9	0.00	–
Diabetes mellitus	0.999	0.00	0.00	–
Mean arterial pressure	0.996	3.7	0.00	2.9
TAPSE/PASP ratio	0.989	0.00	0.00	–

*TAPSE* tricuspid annular plane systolic excursion, *PASP* pulmonary artery systolic pressure

**Fig. 1:**
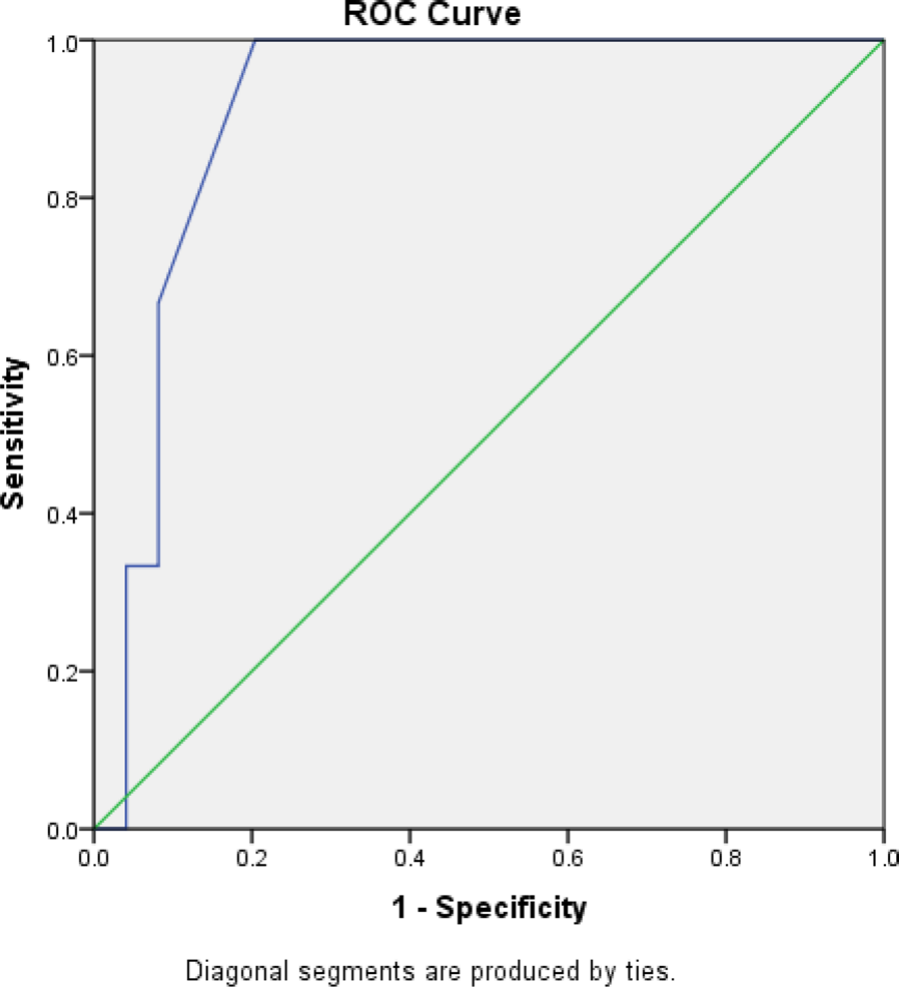
Area under the curve for rehospitalization with heart failure

**Fig. 2:**
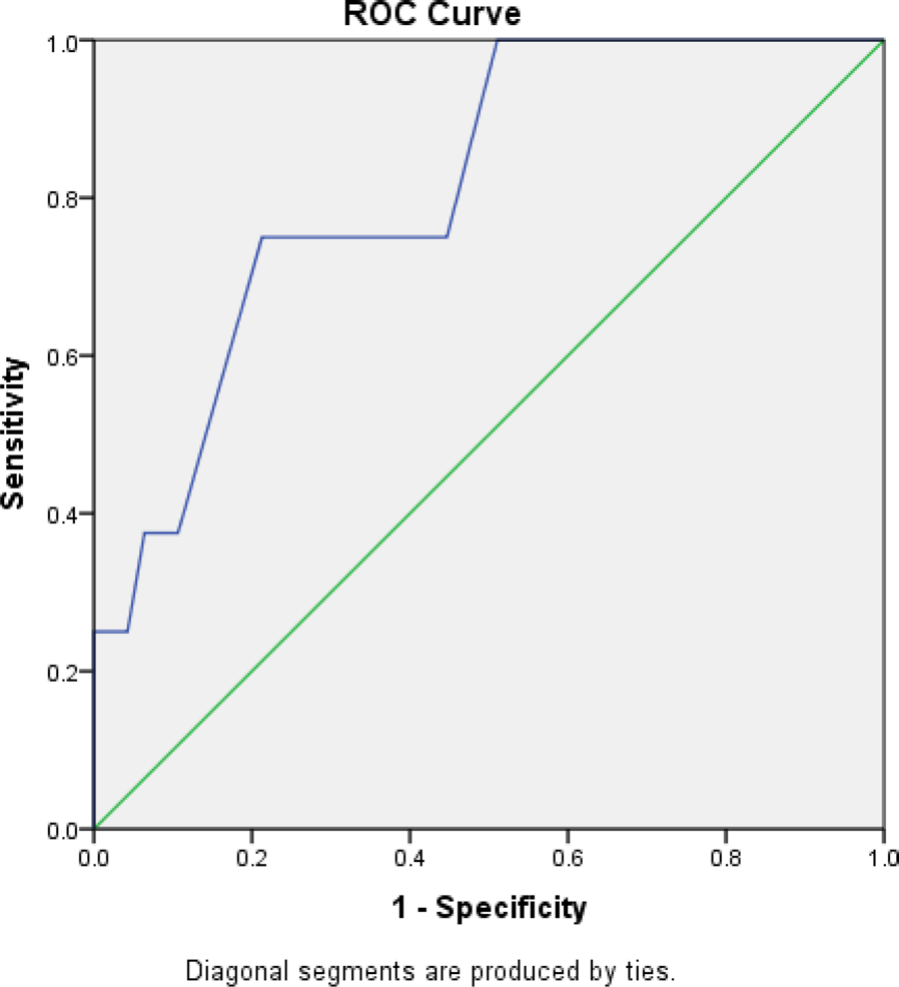
Area under the curve for recurrent pulmonary embolism

ROC curve analysis showed that TAPSE/PASP ratio predicted rehospitalization with heart failure at a cutoff point ≤ 0.325, with 100% sensitivity and 79.6% specificity. It also predicted recurrent pulmonary embolism at a cutoff point ≤ 0.325, with 75% sensitivity and 78.7% specificity (Table 8, Figs. 1, 2).

**Table 8:** ROC analysis for TAPSE/PASP ratio in predicting different outcomes for the patients enrolled with acute pulmonary embolism

Parameters	Cutoff	Sensitivity (%)	Specificity (%)	AUC (95% CI)	*P* value
Rehospitalization with heart failure	≤ 0.325	100	79.6	0.912 (0.834–0.989)	0.001
Recurrent pulmonary embolism	≤ 0.325	75	78.7	0.814 (0.669–0.958)	0.005

## Discussion

The main findings of this study include:

1. Among the recruited patients, 10.9% needed rehospitalization with heart failure, 14.5% developed recurrent pulmonary embolism, and mortality was 9.1% through a period of three months follow up.
2. TAPSE/PASP ratio was significantly lower among patients who developed adverse outcomes.
3. TAPSE/PASP ratio was among the independent predictors of rehospitalization with heart failure, recurrent pulmonary embolism but not mortality at 3-month follow-up.
4. TAPSE/PASP ratio predicted rehospitalization with heart failure at a cutoff point ≤ 0.325, with 100% sensitivity and 79.6% specificity, and predicted recurrent pulmonary embolism at a cutoff point ≤ 0.325, with 75% sensitivity and 78.7% specificity.

Multiple studies validated TAPSE/PASP ratio as a simple, noninvasive echocardiographic method for evaluating RV–PA coupling [3]. TAPSE/PASP ratio has been studied as a predictor of clinical adverse outcomes in multiple patient groups. Among patients with heart failure, TAPSE/PASP ratio was among the important prognostic variables for adverse outcomes [5]. Also, TAPSE/PASP ratio predicted the occurrence of adverse outcomes among pulmonary hypertension patients [6]. In the Global-Tri-Valve registry, among patients with tricuspid regurgitation scheduled for percutaneous intervention either repair or replacement, TAPSE/PASP ratio predicted the occurrence of 12-month all-cause mortality [7]. Also, in the EuroSMR registry that included severe mitral incompetence patients indicated for percutaneous valve replacement [8], TAPSE/PASP ratio was an independent predictor of 24-month mortality. Partner 3 trial showed that TAPSE/PASP ratio independently predicted 2-year composite adverse outcomes among low-risk patients with symptomatic severe aortic stenosis indicated for percutaneous or surgical valve replacement [9].

The value of TAPSE/PASP ratio in predicting adverse outcomes in patients with acute PE is not well studied. In this study, TAPSE/PASP ratio independently predicted the occurrence of adverse clinical outcomes in acute PE patients including rehospitalization with heart failure and recurrent pulmonary embolism at 3-month follow-up. This can be explained by the acute desynchrony between the RV afterload and RV contractility that occurs in acute PE patients due to acute increase in RV afterload unmatched by similar compensatory increase in the RV contractility. This desynchrony is reflected on the TAPSE/PASP ratio. This is concordant with the results of a recent study involving patients with acute PE evaluated multiple echocardiographic parameters that can assess RV–PA coupling. This study showed that TAPSE/PASP ratio was independently associated with adverse events during hospitalization [12]. Also, another recent retrospective study involving acute PE patients showed that TAPSE/PASP ratio predicted the development of 7-day adverse outcomes including mortality and hemodynamic compromise. In this study, a cutoff value of 0.4 for the TAPSE/PASP ratio was identified as the optimal cutoff value for predicting adverse clinical outcome in patients with acute PE [13].

### Limitations of the study

The limitations of the study include that it is a single-center study. Also, TAPSE provides data about the longitudinal rather than the global RV function. RHC is the gold standard for assessment of the PASP; however, in this study we used the echocardiographic assessment.

## Conclusions

TAPSE/PASP ratio is a noninvasive tool that can predict development of early adverse clinical outcomes among patients with acute PE including rehospitalization with heart failure and recurrent pulmonary embolism. Further multicenter studies are needed to validate the use of this bedside parameter to predict adverse outcomes in this critical patient group.

## Data Availability

The datasets used and/or analyzed during the current study are available from the corresponding author on reasonable request.

## Abbreviations

PE: Pulmonary embolism
RV: Right ventricle
PA: Pulmonary artery
TAPSE: Tricuspid annular peak systolic excursion
PASP: Pulmonary artery systolic pressure
LV: Left ventricle
EF: Ejection fraction
RA: Right atrium
FAC: Fractional area change
ROC: Receiver operator characterized
